# Development of a Qigong Used for Insomnia Therapy (QUIT) Program for Improving Sleep Quality and Blood Pressure in Chinese Women With Menopause: Pre-Post Pilot Test of Feasibility

**DOI:** 10.2196/70226

**Published:** 2025-05-02

**Authors:** Sean Huang, Jung Eun Kim, Wen-Wen Li

**Affiliations:** 1 School of Nursing, College of Health and Social Sciences, San Francisco State University, 1600 Holloway Ave, BH383, San Francisco, CA, 94132, United States, 1 4153382368

**Keywords:** Qigong, sleep quality, blood pressure, menopause, Chinese women

## Abstract

**Background:**

Around 20%-50% of Chinese menopausal women experience insomnia, which is associated with elevated blood pressure (BP). Despite this, the population remains understudied. Qigong, a simple form of Chinese exercise, has been shown to improve insomnia and BP but has not been explicitly used to address menopausal symptoms in Chinese women. This study aims to test the feasibility of a Qigong-based intervention in enhancing sleep quality and BP control in this population.

**Objective:**

This study aimed to develop and pilot test the feasibility of a culturally sensitive Qigong Used for Insomnia Therapy (QUIT) intervention in improving sleep quality and BP among Chinese menopausal women.

**Methods:**

From August 2023 to May 2024, this study used a 1-group pretest-posttest design (N=22) to evaluate the QUIT intervention. The intervention consisted of a 10-minute Qigong demonstration video, a 10-minute practice and return demonstration and a 5-minute insomnia counseling session at baseline. Participants were instructed to engage in daily 10-minute Qigong practice for 1 month. Outcome measures, including sleep quality and BP, were assessed at baseline and at the 1-month follow-up. Data on demographics were collected via self-reported questionnaires. At the end of the study, participants were interviewed using semistructured questions to assess their perception of the intervention’s feasibility. Qualitative data were analyzed using content analysis, with interviews transcribed and coded independently by the principal investigator and research assistant. Categories related to feasibility, adherence, and barriers were identified. Quantitative data were analyzed using SPSS 27.0 (IBM Corp), using descriptive statistics and paired sample *t* tests to assess changes in sleep quality and BP, with statistical significance set at .05.

**Results:**

The mean age of participants was 53.78 (SD 8.79, range 42‐74) years. Most participants lived with relatives or friends (20/22, 91%), were employed (16/22, 73%), were married (19/22, 86%), and had at least high school education (19/22, 86%). The mean 23-item Sleep Quality Scale score significantly improved from 18.59 (SD 11.41) at baseline to 15.64 (SD 9.65; mean difference 2.96, SD 7.04; *t*_21_=1.97, *P*=.03) after 1 month, indicating better sleep quality (the 23-item Sleep Quality Scale was reversely scored). There was a trend toward reduced systolic BP from 115.47 (SD 14.95) at baseline to 113.59 (SD 13.93; mean difference −0.89, SD 1.64; *t*_21_=−1.15, *P*=.26) after 1 month. Diastolic BP also improved from 74.69 (SD 10.81) at baseline to 71.41 (SD 16.82) at 1 month (mean difference −3.28, SD 4.04; *t*_21_=−0.81, *P*=.43).

**Conclusions:**

The QUIT intervention was culturally sensitive, low-cost, and easy to implement. It showed significant improvements in sleep quality and trends toward reduced BP in Chinese menopausal women. Further investigation is recommended to further test the QUIT intervention to establish a robust program across different states. Once validated, the QUIT intervention may be implemented in various clinical settings to help Chinese menopausal women achieve optimal sleep quality and BP management.

## Introduction

In the United States, approximately 30% of individuals experience from at least 1 symptom of insomnia [1]. Insomnia is characterized by difficulties falling or remaining asleep, which causes significant distress to the individual [2]. This condition leads to decreased cognitive ability, difficulty functioning, and fatigue and increases the risk of developing cardiovascular disease [3]. Around 20%-50% of women undergoing menopausal transition are affected by insomnia, and the prevalence of the condition is higher in Chinese menopausal women (51%-55%) than in Caucasian (40%), Japanese (28%), and Korean (16%) women [4]. Developing accessible intervention options for chronic insomnia is necessary to combat the disorder in Chinese menopausal women.

Traditional Chinese medicine (TCM) has been successfully used to treat insomnia and alleviate some symptoms, such as fatigue and low energy, in Chinese women [5-7]. TCM has been used for thousands of years as a medical practice to treat disorders such as insomnia. This medical practice is rooted in maintaining a balance between yin and yang, 2 forces that make up the force of life, Qi. Qigong, a very affordable treatment modality, is a common form of Chinese medicine that is used to promote circulation of the brain, which can, in turn, help with sleep and body relaxation at night [8]. However, its effectiveness in Chinese immigrant women has rarely been studied. Literature notes that Chinese immigrants are less compliant with Western medical regimens if their health self-management is influenced by their cultural beliefs and practices, such as TMC [9]. Thus, a sole traditional western health practice may not be feasible in this Chinese women population. Given this, Qigong was used as our primary intervention to improve sleep quality for Chinese menopausal women. Specifically, Ba Duan Jing, a popular and straightforward form of Qigong, was used in our study, which is characterized by 8 slow movements coupled with breathing exercises.

In addition to Qigong, a conventional Western sleep regimen was also used to enhance our therapy for insomnia [10-13]. It asks the participants for several behavioral modifications, which are listed here. (1) Maintaining a consistent sleep schedule is crucial to reducing insomnia as it prepares the brain to transition to sleep. By performing a nighttime routine at the same time every day, the brain is given around 30 minutes to slow down before bedtime [10]. (2) Avoiding caffeine, which is helpful to avoid fitful sleeping. Caffeine taken up to 6 hours before bedtime causes sleep disturbances and a reduced total sleep time [11]. (3) Avoid consuming excessive alcohol close to bedtime, which promotes sleep continuity and decreases wake periods [12]. (4) Following a balanced diet promotes falling asleep faster and maintaining the depth of sleep [13]. (5) Removing distractions or stimulants, such as electronics and bright lights or noises before bedtime, creates a good sleeping environment and primes the brain for sleep [10].

In addition to the traditional Western regimen, we added a Chinese medicine–based regimen, which includes practicing Qigong daily and soaking one’s feet before bedtime. Qigong helps Qi (energy) movement, which supports overall circulation, and practicing it daily helps with consistent sleep quality [14]. Besides Qigong, soaking one’s feet in hot water before bedtime also increases circulation, thus allowing the body to repair and relax. A study found that soaking the feet increased blood flow to the rest of the body, including up to the earlobes, and reduced blood pressure (BP), which benefits cardiovascular health [15].

Since both Qigong and soaking one’s feet may improve sleep quality and reduce BP, it is essential to measure both sleep quality and BP as the outcomes to test the feasibility of the intervention. Insomnia results in activating a sympathetic nervous system, which in turn increases heart rate and metabolic rate [16]. This is interesting because the association between shortened sleep duration and higher BP suggests that treating insomnia can improve BP levels, thereby reducing the risk of hypertension and other health problems. Likewise, high BP is a significant contributing factor for insomnia [17,18]. A meta-analysis suggested that a combination of both insomnia and high BP would put patients at a greater risk of developing cardiovascular disease [17,18]. Thus, managing insomnia and BP can help improve patients’ health conditions [19]. Nevertheless, there is limited evidence to support the feasibility of adopting insomnia treatments to also help improve BP [20,21]. Given the above, developing methods to improve both insomnia and BP can be beneficial in reducing the risk of strokes, heart failure, and other cardiovascular issues in Chinese menopausal women.

The purpose of this study was to develop and pilot test the feasibility of a Qigong Used for Insomnia Therapy (QUIT) program in improving sleep quality and BP in Chinese menopausal women. The QUIT program consists of 2 components: daily Qigong practice and a Western- and Eastern-fused sleep regimen (ie, the aforementioned behavioral modifications).

## Methods

### Design

A 1-group pretest-posttest design was used to test the feasibility of the QUIT intervention. This design allows for assessing whether the intervention works for Chinese women and if there is a trend for improvements in sleep quality and BP.

### Setting

Participants were recruited from a Chinese community health care center in Chinatown, San Francisco. This center serves a large population of Chinese residents (more than 20,000), the majority of whom are first-generation immigrants.

### Sample

In total, 22 Chinese immigrants in the San Francisco Bay Area were recruited to participate in this study. The inclusion criteria were (1) self-identified Chinese immigrant, (2) 45 years of age or older, (3) menopausal, (4) reported to be experiencing from insomnia, (5) can read or speak Chinese (Cantonese or Mandarin). The exclusion criteria were: (1) terminal illness, and (2) cognitive impairment.

### Sample Size

The sample size (N=22) was determined based on the resources available in the study period and the study objectives (with a purpose to pilot test feasibility of the QUIT intervention).

### Recruitment

The research team developed and discussed a recruitment strategy plan. First, a flyer in Chinese was posted in the community health clinic to explain the study’s purpose and provide a toll-free number to discuss the study. For those who made contact and agreed to participate in the study, the research assistants obtained verbal consent and conducted a phone-based eligibility-screening interview, followed by an appointment for an interview visit. A referral system was also used to recruit participants, including word-of-mouth through health care providers and study participants who had already taken part in the study.

### Study Procedure

A total of 2 bicultural and bilingual (Chinese and English) research assistants were recruited and trained by the principal investigator (PI) for one day (8 h) on the study protocol, understanding and implementing the QUIT intervention, interview techniques, and data management and analysis. These 2 research assistants were also trained by a Qigong master (roughly eight 1-h sessions) until they mastered the Qigong practice.

At the initial (baseline) visit, the research assistant explained the study’s purpose, procedures, and requirements and the right to decline to participate and discontinue participation at any time. Written informed consent and a Health Insurance Portability and Accountability Act (HIPAA) authorization were obtained. Participants completed the self-report questionnaire on demographic, cultural, and clinical factors to be used as baseline descriptive data. The participants’ insomnia levels were measured using the 1-item Sleep Quality Scale (SQS-1)- and 23-item Sleep Quality Scale (SQS-23) as baseline data.

The research assistant measured the participants’ BP 2‐3 times using a digital sphygmomanometer, with a 5-minute interval between 2 measurements. A third measurement was performed if the first 2 systolic BP readings differed by 10 mm Hg or more or if the first 2 diastolic BP differed by 5 mm Hg or more. The average BP was calculated from the 2 or 3 readings to get a more reliable BP measurement.

Subsequently, the research assistant showed the Qigong video to study participants, practiced with participants for all 8 movements, and asked participants to return to demonstrate the movements to ensure their correct understanding of how to practice Qigong properly (10 mins). The participant was asked to do Qigong once daily, 7 days per week, for 4 weeks. The participant was also instructed to perform Qigong consistently at around the same time each day and at least 2 hours before bedtime. In addition, the entire QUIT regimen, including the frequency of Qigong and other lifestyle modifications (eg, drinking herbal tea to replace coffee or caffeinated tea), was discussed with each participant(5 mins). Participants were asked to adhere to this regimen for 1 month. The entire initial visit lasted for about 40 minutes.

After the initial visit, 1 follow-up phone call was made at midpoint (2 wk from the baseline visit). During this phone call, the same research assistant (from the initial visit) checked in with the participants to discuss any challenges they experienced while carrying out the regimen. The research assistant then focused on problem-solving these challenges with the participants.

At the second (final) visit (4 wk from the baseline visit), participants were asked to return to measure their BP and insomnia levels using the same Sleep Quality Scale (SQS) questionnaires from the baseline. The BP measurement was conducted in the same manner as the initial visit. After BP measurements were taken and SQS questionnaires were filled out, the research assistant conducted an individual interview with each participant to assess the feasibility of the intervention protocol. The semistructured questions used to assess participants’ feedback included their comprehension of the QUIT content, the usefulness of the QUIT, the ease of following the QUIT intervention protocol, and the strengths and limitations of the QUIT intervention. Sample questions included, “Were you able to comprehend the QUIT instruction? If yes, please explain your answers.”

The interview session was audiotaped with the consent of the participants. The second visit lasted around 45 minutes.

### Measurements

#### Measurement of Sleep Quality

##### SQS-1

The SQS-1 (single item) was used to measure overall sleep quality over a 7-day recall period with a rating from 0 to 10. A higher score indicated better sleep quality [19]. This single-item SQS is more suitable than lengthier sleep questionnaires such as the Pittsburgh Sleep Quality Index (PSQI) to assess sleep quality [19]. Since this instrument has only 1 item, it does not cause any burden for participants and has been used in published studies [22-26], including studies conducted in China [23]. Concurrent criterion validity demonstrated a strong correlation (inverse) between the SQS-1 and PSQI (Goodman-Kruskal correlation=0.92) in populations with insomnia. Test-retest reliability was 0.62 for sleep stability in patients with insomnia over a 4-week period.

##### SQS-23

Besides SQS-1, another insomnia measure, SQS-23, was chosen to help measure different aspects of insomnia. SQS-23 showed good reliability (Cronbach α=0.89) in Chinese patients, and the scoring system is straightforward and easy. The SQS-23 contains 4 factors of sleep quality, including difficulty in getting up (factor 1: 2 items – negatively scored), difficulty in falling asleep (factor 2: 5 items- negatively scored), sleep recovery (factor 3: 6 items - positively scored) –and daytime dysfunction (factor 4: 10 items – negatively scored). Participants used a 4-point Likert scale to report the frequency of their sleep behaviors, with 0 indicating “few,” 1 indicating “sometimes,” 2 indicating “often,” and 3 indicating almost always. A total score was calculated by summing all 23 items, with factor 3 being reverse-scored to match the scoring of the other factors. A higher score indicated poorer sleep quality (possible score range: 0‐69). Sample questions for 4 factors include the following: “Having refreshed feeling of body after sleep” for factor 1; “Difficulty in getting back to sleep after nocturnal awakening” for factor 2; “Regaining vigor after sleep” for factor 3; and “Sleepiness that interferes with daily life” for factor 4.

### Measurement of BP

An Omron brand digital BP machine (code: HEM-7201) was used to measure the patients’ BP. BP was obtained twice, and the values for systolic and diastolic BP were averaged.

### Ethical Considerations

This study protocol was approved by the Institutional Review Board of the San Francisco State University (approval number 2023‐038). All participants provided informed consent before data collection. Participant data were anonymized during analysis to ensure privacy and confidentiality. All data were handled in accordance with institutional data protection guidelines, and permission to tape the interviews was obtained from the institutional review board. At the end of the second visit, a gift card (US $50) was presented to each participant to show appreciation for their participation in the study. This incentive was also approved by the institutional review board.

### Data Management and Analysis

Data on demographic information and the participants’ evaluation of the QUIT content were gathered through self-reported questionnaires filled out by each participant. Open-ended questions were posed to probe the participants’ reactions to the QUIT intervention and the overall visit (tolerance levels, ability to track, clarity, and comprehensibility). Close-ended questions asked participants to fill out relevant health and demographic information and to use a 5-point Likert Scale (extremely unhelpful to extremely helpful) to rate the usefulness of QUIT.

For qualitative data, content analysis was used to systematically examine participants’ answers to the feasibility-related questions described in the “Study Procedure” section. First, 1 of the 2 research assistants transcribed the audiotaped interview data verbatim. The PI and research assistant then independently analyzed the interview data using a structured coding scheme. The analysis was based on extensive line-by-line coding of the raw data. Coding and categorization continued until no new information was forthcoming, and the categories appeared “saturated.” The percentage of agreement on coding categories between the PI and research assistant was calculated. Any disagreements were then discussed, and further data analysis was performed. It was ensured that the coding between the PI and research assistant reached at least 80% agreement. The final categories (themes showing the perceived strengths of the QUIT intervention) are listed in Textbox 1. These categories are related to the feasibility and adherence of the QUIT intervention. The areas for improvement of the QUIT intervention are also presented. This information is anticipated to help guide the refinement and optimization of the QUIT intervention.

For quantitative data, SPSS 27.0 was used to analyze and describe baseline sociodemographic and clinical data. Data entry and management were done by one research assistant and verified by the PI. Descriptive statistics were used to screen data for missing values and outliers and to describe the demographic and clinical variables. A paired sample *t* test was used to examine the difference between pre- and postintervention in terms of changes in sleep quality and BP. Statistical significance was set at .05.

Textbox 1. **Strengths of Qigong Used for Insomnia Therapy (QUIT) in promoting well-being.**

 **Easy and convenient treatment plan**

- It is easy to practice Qigong and follow a sleep regimen
- Qigong can be done anywhere and at any time
- Having a routine makes life more organized

**Improvement of sleep quality**

- One falls asleep more quickly
- It is easier to fall asleep by reducing thoughts of daytime thinking and activities

**Promotion of breathing and relaxation**

- It helps to breathe
- It encourages practicing breathing exercises
- It creates a peaceful mood
- It lets the body become more relaxed

**Improvement of physical health**

- Helps with losing fat
- Increases flexibility of body

**Improvement of mental health (positive mood or mind)**

- Clears mind
- Elevates mood

**Improvement of energy**

- Improves energy
- Increases motivation to do one’s daily activities

**Increased exercise amount**

- Practicing Qigong helps one exercise regularly
- It serves as another form of exercise on top of a regular workout routine

## Results

### Sample Characteristics

All participants were women (n=22). The mean age of participants was 53.78 (SD 8.79; range 42‐74) years. The majority of the participants lived with relatives or friends (20/22, 91%), were employed (full-time or part-time job: 16/22, 73%), were married (19/22, 86%), and had at least high school education (19/22, 86%; Table 1).

**Table 1.** Baseline characteristics of Chinese menopausal women (N=22).

Variables		Values
Age, mean (SD)		53.78 (8.79; range 42‐74)
Gender (female), n (%)		22 (100)
Education, n (%)		
	Primary school	2 (9.1)
	Middle school	1 (4.5)
	High school	2 (9.1)
	Associate	4 (18.2)
	Bachelor	5 (22.7)
	Master and above	8 (36.4)
Marital Status, n (%)		
	Married	19 (86.5)
	Divorced or separated	1 (4.5)
	Single	1 (4.5)
	Widow	1 (4.5)
Religion, n (%)		
	None	10 (45.5)
	Buddhism	4 (18.2)
	Taoism	2 (9.1)
	Christian	3 (13.6)
	Others	2 (9.1)
	Missing	1 (4.5)
Are you living with your family or friends(s)?, n (%)		
	No	2 (9.1)
	Yes	20 (90.9)
Employment, n (%)		
	Full time	14 (63.6)
	Part time	2 (9.1)
	Not working	2 (9.1)
	Retired	3 (13.6)
	Others	1 (4.5)
Annual income (US $), n (%)		
	Less than or equal to $9,999	3 (13.6)
	$10,000‐99,999	2 (9.1)
	$20,000‐29,999	4 (18.2)
	$30,000-$39,000	2 (9.1)
	$40,000-$49,999	1 (4.5)
	$50,000-$59,000	2 (9.1)
	>$100,000	3 (13.6)

### Feasibility Evaluation of the QUIT Intervention

#### Overview

This study evaluated the feasibility of the QUIT intervention by gathering both quantitative and qualitative feedback from participants. Using a 5-point Likert scale (1=extremely unhelpful or inappropriate; 5=extremely helpful or appropriate), participants rated the overall helpfulness and appropriate content of the intervention. More than half (12/22, 55%) found the intervention to be helpful or extremely helpful, with 50% (n=11) rating it as helpful and 5% (n=1) as extremely helpful. Notably, no participants rated the intervention as unhelpful or extremely unhelpful, though 45% (10/22) remained neutral. Regarding the appropriateness of the content, the response was even more favorable, with 82% (18/22) of participants considering the content to be appropriate or extremely appropriate—specifically, 41% (9/22) rated it as appropriate and another 41% (9/22) as extremely appropriate. Only 18% (4/22) remained neutral, and no participants found the content to be inappropriate. In addition to these quantitative findings, qualitative feedback was collected to explore the perceived strengths of the QUIT intervention and identify areas for improvement, which provided valuable insight into participant experiences and guided future enhancements of the intervention.

#### Strengths of the QUIT Intervention

##### Ease and Convenience Treatment Plan

Many participants thought implementing the routine was easy and convenient. The routine consisted of a set of movement practice and behavioral changes that did not require any additional equipment. One participant claimed it was “easy” since they could do it “anywhere, anytime.” Another participant said the routine was “stable and easily established,” while others said it made their “life more organized.”

##### Improvement of Sleep Quality

Quite a few participants commented that the Qigong video helped assist them in falling asleep easier and faster. One participant said that “practicing Qigong decreases the time needed to fall asleep” and “helps me fall asleep faster.” Another participant explained that it became easier to fall asleep by reducing thoughts of daytime thinking or activities.” Other participants added that the routine helped them “sleep deeper.”

##### Promotion of Breathing and Relaxation

Many participants found that the Qigong exercise helped regulate their breathing. One participant said that it “helps me breathe,” while another observed that it “fixes my breathing.” Another participant noted that the routine was “good exercise to have every day” since it “allows [them] to practice breathing.” Some participants claimed that the routine “let my body become more relaxed.”

##### Improvement of Physical Health

Several participants reported that the QUIT intervention (sleep hygiene routine) positively improved their physical health. Some participants said that establishing a good sleep routine “can help with losing fat.” Some reported that it gave them greater flexibility, enabling them to “move my arms around higher,” after practicing Qigong.

##### Improvement of Mental Health (Positive Mood or Mind)

Several participants noted an uplift in their mood after practicing the Qigong routine. One participant mentioned that it “clears my head,” while another shared that they felt “in a more peaceful mood.”

##### Elevated Energy Levels

The participants commented that the Qigong practice helped them to become more energetic during and after the practice. The participants commented that Qigong “improves in terms of energy levels” and “I feel more energetic and motivated to do my daily activities.”

##### Development of Exercise Habits

The participants appreciated the additional Qigong exercise alongside their regular workouts. One participant noted that practicing Qigong “helps me to exercise regularly,” even though they had not done so before. Another mentioned it as “another form of exercise on top of my regular workout, such as jogging.”

### Areas of Improvement of the QUIT Intervention

A couple of patients complained it is hard for them to practice Qigong daily. Some quotes included “I cannot do this every day,” “it is hard for me to do it consistently,” and “having to do this every day is challenging for me.”

### Pilot Test of the Outcomes of the QUIT Intervention

#### Changes in Sleep Quality Before and After Intervention

Sleep quality, measured using SQS-1, significantly improved from 6.68 (SD 2.42; baseline visit) to 7.36 (SD 2 postintervention; *t*_21_= 2.19, *P*=.04*;* Table 2). The SQS-23 (negatively [reversely] scored) sleep quality score was also significantly improved from 18.59 (SD 11.41) to 15.64 (SD 9.65); *t*_21_= 1.97; *P*=.03*;* Table 2).

**Table 2.** Changes in outcomes from baseline to 1 month.

Outcomes	Baseline (N=22)	1 Month (N=22)		Mean change	
	Mean (SD)	Mean (SD)	Mean difference (SD)	*t* test (*df*)	*P* value^a^
SQS-1^b^	6.68 (2.42)	7.36 (2.00)	0.68 (1.46)	2.19 (21)	.04
SQS-23^c^ (reversely scored)	18.59 (11.41)	15.64 (9.65)	2.96 (7.04)	1.97 (21)	.03
Systolic BP^d^	115.48 (14.95)	113.59 (13.93)	−0.89 (1.64)	−1.15 (21)	.26
Diastolic BP	74.69 (10.81)	71.41 (16.82)	−3.28 (4.04)	−0.81 (21)	.43

a Statistical significance was set at *P*<.05.

b SQS-1: 1-item Sleep Quality Scale.

c SQS-23: 23-item Sleep Quality Scale.

d BP: blood pressure.

#### Changes in BP Before and After Intervention

The baseline systolic BP was 115.48 (SD 14.95) mm Hg, and diastolic BP was 74.69 (SD 10.81) mm Hg (Table 2). Approximately 4 weeks after the baseline visit (postintervention), the average systolic and diastolic BP dropped to 113.59 (SD 13.93) mm Hg and 71.41 (SD 16.82) mm Hg, respectively (Table 2). Although the data were not statistically significant, both systolic BP (−0.89, SD 1.64 mm Hg; *t*_21_=−1.15; *P*=.26) and diastolic BP (−3.28, SD 4.04 mm Hg; *t*_21_=−0.81; *P*=.43) showed a trend toward improvement from baseline to week 4 (postintervention; Table 2).

## Discussion

This study outlines the development of a QUIT intervention approach in Chinese menopausal women with insomnia to increase sleep quality. Of the 22 participants, most had higher education, did not practice religion, lived with family or friends, were employed full-time, and were married.

### Principal Findings

#### Feasibility Evaluation of the QUIT Intervention

Overall, the participants found the QUIT intervention very clear and easy to understand. Most participants could follow the instructions without difficulty and had no problem executing the exercises demonstrated in the video. The strengths of the Qigong routine include its easy implementation and the inclusion of familiar, culturally appropriate movements that helped participants ease into the routine. Participants also appreciated that the Qigong routine could be performed at any time and place, and they recognized the positive impacts it had on their lives. In addition, participants reported improvements in sleep quality and other aspects of physical and mental health. Thus, the QUIT intervention was feasible and culturally appropriate for Chinese menopausal women.

Areas for improvement were also identified through participant feedback. The most challenging aspect mentioned was the difficulty of practicing Qigong every day. To address this, future refinements of the QUIT intervention could consider reducing the frequency of Qigong practice from daily to 4 or 5 days per week.

#### Pilot Test of the Outcomes of the QUIT Intervention (Insomnia and BP)

The results demonstrated that the QUIT intervention resulted in statistically significant improvement in sleep quality 18.59 (SD 11.41) to 15.64 (SD 9.65); reversely scored, indicating higher scores reflect poorer sleep quality (mean difference 2.96, SD 7.04; *t*_21_=1.97, *P=*.03) and a trend in improving systolic (mean 115.47, SD 14.95 to mean 113.59, SD 13.93; *t*_21_=−1.15, *P=*.26) and diastolic BP (mean 74.69, SD 10.81 to mean 71.41, SD 16.82; *t*_21_=−0.81, *P*=.43). Both sleep quality outcomes (SQS-1 and SQS-23) showed significant improvement, indicating the feasibility of the QUIT intervention in improving insomnia among Chinese women experiencing menopause. For further comparisons with other studies, the data on SQS-23 will be used, as it presents various aspects of sleep patterns and quality.

### Comparison to Previous Work

Our finding in improving sleep quality is similar to a randomized controlled trial (RCT) conducted in China that tested the effects of Tai Chi on sleep quality [27]. This study found that practicing Tai Chi for 60 minutes, 3 times a week for 8 weeks, significantly improved overall sleep quality compared to the control group (*t*=2.05, *P*=.04) (from mean 6.08, SD 4.01 to mean 4.89, SD 4.43; reversely scored) [27]. Our QUIT intervention had less intensity (10 min of Qigong practice daily for a month) than this RCT, but our study found a significant improvement in sleep quality similar to Wang et al [27]. However, since our sample size is small and there was no control group, further testing of our QUIT intervention is required to investigate its efficacy. The reason that our less intense intervention (10 min instead of 60 min per session) had a similar result to this RCT is that we implemented the Qigong practice on a daily basis, which helped participants treat it as a routine so they would not forget to do Qigong, generating a significant effect in improving their sleep quality.

It is noteworthy that there is a similarity between Qigong and Tai Chi [28]. Both are ancient Chinese practices that focus on the cultivation and balance of Qi (life energy) through coordinated movements, breathing techniques, and meditation. They aim to improve physical health, mental clarity, and emotional balance. In addition, both practices emphasize relaxation, slow movements, and mindfulness. The difference is that Qigong focuses on cultivating and balancing Qi through simple, repetitive movements, breathing techniques, and meditation, while Tai Chi, a martial art, involves more complex, flowing sequences of movements [27]. In our QUIT study, we chose a simpler form to promote daily practice.

Another study conducted in Spain examined the effects of Qigong on 125 postmenopausal women and showed a significant improvement in overall sleep quality [6]. The results, measured with the PSQI questionnaire, indicated significant improvements in sleep quality in the intervention group. Participants completed 24 sessions over 12 weeks (two 60-min sessions weekly). The overall sleep quality score (reversely scored) improved from mean 7.56 SD (4.56) to mean 5.89 (SD 3.74; *F*_1.115_=12.27*, P*=.001), with a mean difference of 1.67. The score ranged from 0 to 21.

In comparison, the study from Spain had a longer, less frequent intervention (24 sessions over 12 wk, with two 60-min sessions weekly) compared to our QUIT intervention, which involves a shorter, daily practice (eg, 10 min daily for 1 month). Both studies showed statistically significant improvements in sleep quality (mean difference of 1.67 in Spain’s study vs 2.69 in our QUIT study). This finding may suggest that frequent shorter practice has the same effect as less frequent longer sessions. Based on interviews with some of our study participants, they commented that practicing 10 minutes per session may be difficult to adhere to, and a 60-minute session would be even harder. This would create more barriers for Chinese women in maintaining the Qigong practice long-term. Thus, it is recommended that future studies test the feasibility and efficacy of a shorter duration of Qigong practice, such as less than 10 minutes, for 4 to 5 days a week in Chinese menopausal women to establish more robust results.

It is noted that compared to Wang et al [27] (the study in China) and Carcelén-Fraile et al [6] (the study in Spain), our results on the overall improvement of sleep quality are more promising. This difference may be attributed to behavioral modifications included in our QUIT regimen. Participants of the QUIT intervention made additional changes to their lifestyle, such as avoiding coffee and alcohol and soaking their feet in hot water in addition to performing the daily qigong exercise, which may explain the increase in sleep quality scores.

Regarding reduction of BP, compared to a study conducted in Hong Kong which tested the effect of Tai Chi in reducing BP [29], our study showed improvement in both systolic BP (mean difference from baseline to week 4: −1.89) and diastolic BP (mean difference −3.28 from baseline to week 4); but the study by Ko et al [29] only showed improvement in systolic BP but no change in diastolic BP (−6.0 mm Hg for systolic and 0.00 mm Hg for diastolic BP). In general, both studies showed promising results of their intervention. There was a similarity between our Qigong session and the study by Ko et al [29]. Both interventions use movement techniques to promote Qi movement [28], which in turn improves BP. The difference is that our approach used video instruction for Qigong practice, which is very economical and requires less labor so that it could be practiced more frequently, such as 5-7 days per week. In contrast, Ko et al [29] asked their participants to practice Tai Chi with a Tai Chi master in person twice (1 hour each time) every week, which is more expensive and labor intensive [29]. If both interventions can improve BP, a low-cost and sustainable intervention should be preferred in the clinical setting as it will be easier for self-management of BP at home without economic and physical limitations (eg, patients do not need to travel to practice Qigong and can practice Qigong at any time and any place). In the future, it is recommended to use video instruction instead of hiring a master to teach twice per week to be further investigated for its long-term feasibility and efficacy.

### Strengths

The QUIT intervention is a potentially affordable approach, using a 10-minute Qigong video as the primary source of health education. This is followed by a short 10-minute practice and return demonstration of Qigong and a 5-minute discussion of the sleep regimen by trained personnel. It is easy to implement, so it is anticipated that the QUIT intervention can potentially be applied in the clinical setting once it is tested to be effective in a larger population of menopausal Chinese women in the future.

### Limitations

The study limitations include possible participant bias and threats to the design’s validity. First, Chinese women tend to overemphasize improvements to their health and adherence to medical advice to please their health care providers. This may result in discrepancies between self-reported and genuine health improvements. To mitigate this, the participants were told to report the true results as much as possible to help us generate reliable results. In the future, sleep quality could be assessed by using other methods, such as a device to measure sleep quality in an objective way, to compare the results between self-report and objective measurements.

Second, a threat to our design’s validity is the uncertain effect of the QUIT intervention. It is not certain that adherence to the QUIT intervention was solely responsible for improvements in sleep quality and BP. For example, discussions with health experts and encouragement to make behavioral changes may have motivated participants to adopt other lifestyle changes beyond what was outlined in the QUIT regimen (eg, use of sleep assist devices). In this study, the participants were informed to follow the QUIT protocol and no other treatment regimen during the study period, but they were not monitored 24/7, so it is impossible to completely stop them from doing something beyond what was advised. It is recommended that in the future the QUIT study be replicated and conducted with a 2-armed (intervention and control groups) RCT to eliminate confounding effects.

### Implications for Clinical Practice

The results of our QUIT intervention approach may prove to be a culturally suitable method for treating insomnia and improving BP in Chinese menopausal women. Given the current lack of affordable and accessible treatment options (eg, shortage of medical practitioners) for both insomnia and BP, QUIT illustrates the potential for simple habitual changes to improve Chinese women’s health outcomes. One advantage of QUIT is its simplicity. After a single training session with an expert, patients can practice the QUIT regimen at home at their convenience. Thus, implementing the QUIT intervention may be clinically meaningful and promising for Chinese menopausal women.

### Conclusions

Our pilot QUIT intervention built upon the existing recommended sleep regimen (eg, adhering to a healthy diet) to promote blood circulation, providing both a feasibility test and assessment of the QUIT intervention, to shed light on the potential direction for the future research, and to help develop a robust culturally sensitive intervention to improve sleep quality and BP in Chinese menopausal women. Our results demonstrated that our QUIT intervention was feasible and culturally acceptable for Chinese menopausal women. It is noteworthy that our Eastern- and Western-fused sleep regimen (eg, soaking one’s feet in hot water) along with daily Qigong practice significantly improved sleep quality and may further improve BP over time. Once the QUIT intervention is shown to be effective in enhancing sleep quality and improving BP through more robust studies, the program can be used in Chinese communities.

## Abbreviations

BP: blood pressure
HIPAA: Health Insurance Portability and Accountability Act
PI: principal investigator
PSQI: Pittsburgh Sleep Quality Index
QUIT: Qigong Used for Insomnia Therapy
RCT: randomized controlled trial
SQS: Sleep Quality Scale
SQS-1: 1-item Sleep Quality Scale
SQS-23: 23-item Sleep Quality Scale
TCM: Traditional Chinese medicine
